# Novel Photothermal Graphene-Based Hydrogels in Biomedical Applications

**DOI:** 10.3390/polym16081098

**Published:** 2024-04-15

**Authors:** Alexa-Maria Croitoru, Denisa Ficai, Anton Ficai

**Affiliations:** 1 Research Institute of the University of Bucharest (ICUB), University of Bucharest, Spl. Independentei 91-95, 0500957 Bucharest, Romania; alexa_maria.croitoru@upb.ro; 2 Department of Science and Engineering of Oxide Materials and Nanomaterials, Faculty of Chemical Engineering and Biotechnologies, National University for Science and Technology Politehnica Bucharest, Gh. Polizu St. 1-7, 011061 Bucharest, Romania; denisa.ficai@upb.ro; 3 National Centre for Food Safety, National University for Science and Technology Politehnica Bucharest, Spl. Independentei 313, 060042 Bucharest, Romania; 4 National Centre for Micro- and Nanomaterials, National University for Science and Technology Politehnica Bucharest, Spl. Independentei 313, 060042 Bucharest, Romania; 5 Academy of Romanian Scientists, 3 Ilfov Street, 050045 Bucharest, Romania

**Keywords:** graphene-integrated hydrogels, photothermal therapy, photothermal drug delivery, biomedical applications

## Abstract

In the last decade, photothermal therapy (PTT) has attracted tremendous attention because it is non-invasive, shows high efficiency and antibacterial activity, and minimizes drug side effects. Previous studies demonstrated that PTT can effectively inhibit the growth of bacteria and promotes cell proliferation, accelerating wound healing and tissue regeneration. Among different NIR-responsive biomaterials, graphene-based hydrogels with photothermal properties are considered as the best candidates for biomedical applications, due to their excellent properties. This review summarizes the current advances in the development of innovative graphene-based hydrogels for PTT-based biomedical applications. Also, the information about photothermal properties and the potential applications of graphene-based hydrogels in biomedical therapies are provided. These findings provide a great potential for supporting their applications in photothermal biomedicine.

## 1. Introduction

In present-day medicine, novel, controlled, on-demand stimuli-responsive systems are necessary to be developed in order to enhance the efficacy and controllability of smart materials and to prevent undesirable effects in biomedical applications. Smart materials have physico-chemical properties that can be tuned using internal or external stimuli such as light, pH, temperature, electric or magnetic field, etc. Thus, they show many advantages compared to conventional materials that cannot adapt to the changing therapeutic needs [1,2,3].

In this regard, in the last decade, PTT has gained major interest as a potential treatment strategy for numerous therapies such as cancer therapy, tissue and bone regeneration, wound repair, drug delivery, etc. [4,5]. Compared to traditional therapies, PTT is considered to be suited for biomedical applications because it causes negligible damage, has high penetrability, can be used as an antibacterial treatment, and exhibits minimum side effects. Besides the antibacterial effect, NIR laser irradiation has a role in the personalized release of photothermal agents (PTAs) in the tissue of interest [6,7,8].

In the last decade, modern techniques were used in order to obtain novel and improved bioactive materials (smart materials) that can be envisioned as a new promising class of PTAs. Among many NIR-responsive biomaterials that have been developed, such as sponges, films, nanofibers, and cotton wool, hydrogel-based dressings are considered as best candidates for tissue regeneration and disease therapies, due to their multifunctional properties. Hydrogels have a three-dimensional (3D) porous structure with unique characteristics such as hydrophilicity, swelling, micro/nanosized pores, softness, and the capability to mimic the extracellular matrix (ECM) [9,10,11,12]. Moreover, hydrogels have excellent biocompatibility and good mechanical and degradation properties [13,14]. Their internal micro/nanopores allow the diffusion of small molecules from and to their surroundings [15,16,17].

Many research studies have been conducted on PTT therapy to develop hydrogel-modified composites with great advantages including enhancing the efficacy of photothermal therapeutics [18]. Nevertheless, hydrogels have a major drawback, i.e., a low mechanical strength, which is a challenge for their use in therapeutic applications. To overcome this issue, the functionalization of hydrogels with different nanomaterials can improve specific properties of the materials including thermal, electrical, mechanical, antibacterial, and also biocompatibility. Hydrogels obtained by combining biomaterials with biocompatible PTA can reduce biological toxicity and make them more biocompatible [10,19,20].

Moreover, the loading of different therapeutic agents (natural and/or synthetic) in such structures can help in increasing the healing process and also help in the regeneration of the damaged tissue [21,22,23].

Recent studies have focused their attention on modifying the external temperature that influence the responses of bacteria, cells, and tissues [24,25]. By changing the concentration, irradiation time, and laser intensity of PTA, the target temperature of photothermal hydrogels can be modified. Thus, a low level of local heat between 41 °C and 43 °C can stimulate cell proliferation, angiogenesis, and wound healing. A moderate level of heat between 45 °C and 50 °C leads to minor damage to normal tissue cells but total damage to tumor cells. For infected wound healing, hyperthermia (>50 °C) can effectively inhibit the proliferation of bacteria. Therefore, the change in temperature can control the photothermal effects of hydrogels in order to be used in different biological treatment strategies [13,26].

Among the various NIR-responsive nanomaterials [27,28,29,30,31], graphene-based nanomaterials possess superior optical absorption and photothermal conversion, being used as a powerful PTA. Among the different types of graphene, graphene oxide (GO) is one of the most studied materials for photothermal application, due to its particular physico-chemical properties, such as a large specific surface area, abundant oxygen functional groups (the ability to interact with various biologically active agents), good biocompatibility, thermal stability, etc. [32,33,34]. Thus, GO can absorb NIR radiation and can also be applied for quickly releasing therapeutic agents from the surface through NIR. Owing to the aforementioned characteristics of hydrogels and GO, there are increasing number of scientific studies on the investigation of GO-based hydrogel nanocomposites for NIR-mediated PTT [6,35,36]. For example, Zhang et al. [37] demonstrated that PVA hydrogel incorporated into rGO/MoS_2_/Ag_3_PO_4_ composites exhibited great antibacterial properties against *Staphylococcus aureus* and *Escherichia coli* under NIR irradiation, with 97.8% and 98.33% antibacterial rates within 10 min.

This review aims to discuss the current advances in the development of innovative graphene-based hydrogels for PTT-based biomedical applications. It also provides information about new strategies used for enhancing their structural integrity, functionality, and mechanical properties and performance in different therapies. Photothermal GO hydrogels showed great applications in cancer therapy, wound healing, and bone regeneration, as well as in other biomedical fields. Furthermore, challenges and future perspectives are discussed for designing graphene-based composite hydrogels for use in clinical applications. Based on this information, the potential in vitro and in vivo applications of hydrogels were presented, demonstrating how the physico-chemical or biological responses influence and determine their use in different biomedical fields.

## 2. Hydrogel Classification and Physico-Chemical Property

**Figure 1:**
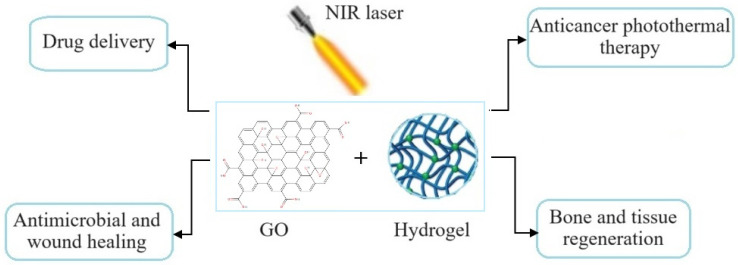
Schematic representation of graphene-based hydrogel applications in different photothermal therapeutics.

Regarding the classification of hydrogels, they are divided into two classes: natural hydrogels and synthetic hydrogels. Although natural hydrogels (e.g., alginate, chitosan, collagen, gelatin, etc.) possess good biodegradability, they have poor mechanical properties and stretchability. On the other hand, synthetic hydrogels (e.g., PEG derivatives, polycaprolactone (PCL), polyvinyl alcohol (PVA), etc.) present better mechanical properties but have poor biological activity and biocompatibility [38]. Moreover, the classification of hydrogels depends on the crosslinking method (physical, chemical, etc.), the ionic charge (cationic, anionic, and neutral hydrogels), and their response to stimuli (temperature, pH, light, magnetic and electric fields, etc.) (Figure 1) [39].

Due to their structure and hydrophilic groups, hydrogels possess many advantages such as mechanical strength, biocompatibility, biodegradability, swellability, and stimuli sensitivity. The swelling property is the most significant one and takes place in three steps: the diffusion of water into the hydrogel network, the relaxation of polymer chains, and the expansion of the hydrogel network. Depending on the crosslinker involved, the hydrogels are normally degradable [40].

## 3. Fabrication of Graphene-Based Hydrogels

Currently, hydrogels have been studied and used in various biomedical applications because of their diverse physico-chemical, biological, and structural characteristics [9]. In recent years, in order to fabricate graphene-based hydrogels, different methods have been used in order to obtain a well-dispersed and interconnected graphene network. The hydrogels can be obtained using physical or chemical crosslinking methods, as well as in situ reduction. When the physical process is used, different mechanisms are involved, such as hydrophobic association, chain aggregation, crystallization, and hydrogen bonding, and the obtained hydrogels are reversible gels. When chemical process is used, chemical covalent bonds are formed, and the obtained hydrogels are permanent and irreversible [41]. The resulting structures exhibit a high surface area, porosity, high electrical and thermal conductivities, an improved mechanical strength, and a tunable pore size distribution, which are critical for biomedical applications such as tissue engineering, wound healing, etc. [42,43].

### 3.1. Physical Crosslinking Approach

This process is the simplest and widely applied in numerous studies. For graphene/hydrogel incorporation, physical interactions such as hydrophobic interactions, π–π stacking, hydrogen bonding, self-assembly, or crystallization take place. When self-assembly method is used, the obtained materials present characteristics, such as high thermal stability, a porous structure, and an ultra-low density [44]. Furthermore, another simple method of forming physical crosslinks is crystallization that occurs after repeated freezing and thawing cycles under certain conditions. For example, Xie et al. [45] designed GO-reinforced cellulose/polyvinyl alcohol hydrogels using a repeated cooling and de-icing method. During these processes, the obtained solution of GO/RCE (stored at −12 °C for 12 h) was mixed with the PVA solution using ultrasonic stirring. After that, the mixture was cast into plastic molds, followed by the freeze–thaw cycles. Haniff and colleagues created a novel antibacterial hydrogel using polyvinyl alcohol (PVA), graphene-based material (GBM), and aloe vera extract (Av) through the freeze–thaw process. The PVA/GBM/Av hydrogel exhibited superior hydrophilicity, electrical conductivity, and stability in phosphate-buffered saline [46]. A recent study demonstrated a good antibacterial activity and enhanced mechanical properties of a GO/rose Bengal/PVA hydrogel prepared by the freezing and thawing method [14].

Carboxymethyl cellulose (CMC) is a type of polymer that can form hydrogels with remarkable biocompatibility, biodegradability, and low toxicity. CMC also shows a good coordination ability with metal ions. By the addition of graphene-based materials, the mechanical properties of the CMC hydrogels and also their ability for adsorption of metal ions could significantly increase. In this regard, Monireh et al. [47] developed biodegradable CMC/GO hydrogel nanocomposite beads physically crosslinked with FeCl_3_.6H_2_O for the selective drug release of doxorubicin (DOX). Also, DOX was loaded on the GO surface through the π–π stacking interaction.

In conclusion, the crosslinked hydrogel synthesis is a simple conventional, easy to fabricate method, but it also has some drawbacks such as low mechanical properties and the irregular dispersion of GO [42].

### 3.2. Chemical Crosslinking Approach

In this method, a proper chemical crosslinker in a suitable solvent is used in order to obtain the 3D network of GO-based hydrogels. This technique is considered a “one-step” method with many advantages, such as low cost, fast, and efficient, especially in the absence of any equipment. A few examples of crosslinkers include glutaraldehyde, glyoxal, 1,4-butanediol diglycidyl ether, dialdehyde starch, N,N′-methylene bisacrylamide, etc. [48,49,50]. In addition, the crosslinking of carbon-based hydrogels can help in preventing the crushing of the hydrogel during the swelling process, leading to improved mechanical properties and great physical integrity of these materials. Graphene-incorporated hydrogels are characterized by their stability and extended degradation time [51].

**Figure 2:**
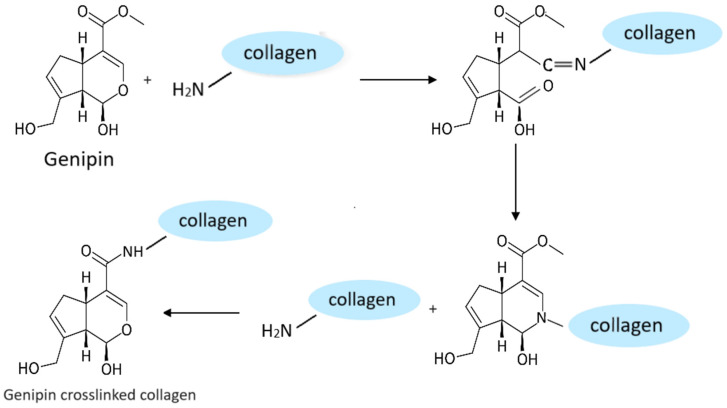
Crosslinking reaction of collagen with genipin.

Unfortunately, these chemical crosslinkers are mainly toxic to the cells of the human body; therefore, the hydrogels must undergo purification processes to remove any unreacted toxic compounds. An alternative for obtaining materials with much lower cytotoxicity or even non-cytotoxic materials is using crosslinking agents of a natural origin, such as genipin (e.g., of crosslinking reaction between collagen and genipin is showed in Figure 2), vanillin, citric acid, tannic acid, and epigallocatechin gallate.

Other disadvantages of chemical crosslinking methods are that these are considered to produce non-homogeneous hydrogels that have poor absorption properties. Although specific enzymes can also be used in the chemical crosslinking process, the cost of the enzymes and the difficulty to produce them represent other disadvantages [52,53]. Vlasceanu et al. [54] developed a new chitosan/gelatin (CS/Gel) hydrogel, reinforced with GO, by using genipin as a crosslinker. An adequate amount of GEL was dissolved in different GO aqueous dispersions (0.5, 1, 2 and 3 wt%). Afterward, GEN solution was added in order to crosslink the CS/Gel/GO hydrogels. The mixture was poured into Petri dishes and left so that the solvent evaporates. The obtained composites were subjected to physico-chemical analyses, and the results showed that GO can be used as an agent for improving roughness and the chemical stability of the polymeric hydrogels.

The synergistic effect and mechanical strength of CS hydrogels were investigated by adding glutaraldehyde (GA) as a chemical crosslinker and by physical crosslinking CS hydrogel with GO nanosheets. At first, the different GO suspensions (0.1 and 0.5 wt%) were obtained, and then, CS powder and acetic acid were added, and the obtained solutions were mechanically stirred overnight. Finally, GA was added in the resultant suspensions. The physico-chemical characterizations demonstrated enhanced mechanical properties and increased stiffness in the CS/GO hydrogels [55].

### 3.3. In-Situ Polymerization

Polymerization occurs on the surface of GO and consists of the conversion of monomers into polymers through localized chain reactions. It has many advantages, such as low energy cost and fast preparation, as well as the good dispersibility and uniformity of GO. Different monomers are used for obtaining GO-based hydrogels: acrylamide, polyacrylamide, N-substituted acrylamides, methacrylate, pyrrole, glycols, etc. Li et al. [56] developed graphene oxide/poly(acrylic acid) hydrogels by in situ polymerization using polyacrylamide monomers. The obtained GO hydrogels demonstrated good electrical conductivity and resistance change upon stretching. Zhang et al. [57] used the in situ polymerization of the monomer polyacrylamide (PAM) and the co-crosslinker N,N-methylene bisacrylamide to fabricate GO/polyacrylamide composite hydrogels. By grafting PAM onto the GO nanosheets, the composite hydrogels displayed good mechanical properties and an extraordinarily high strength. Different preparation methods for graphene-integrated hydrogels are presented in Table 1.

## 4. Role of Crosslinking Agents in Formation of Graphene-Based Hydrogels

There are many types of crosslinking agents (also known as gelators), that play an important role in the formation of graphene-based hydrogels. They are responsible for creating a stable and porous structure through various mechanisms by crosslinking graphene-based materials and forming the interconnected network of the hydrogel. Various binders such as natural or synthetic polymers, small organic molecules, or metal ions can be used and can influence the gel’s structure and properties, such as porosity, mechanical strength, or water absorption capacity [60,61,62].

These characteristics can also be influenced by the concentration of the gelator and the crosslinking conditions. Few common examples of crosslinking agents used for designing graphene-based hydrogels are CS, PVA, polyethylene glycol (PEG), gelatin, etc. These polymers have the ability to form strong crosslinks with the graphene materials, resulting in a stable gel matrix with desirable properties [63,64,65]. For example, GO/CS system was obtained by the amide reaction that significantly improved the mechanical properties of the composite [61].

The GO/CS system can be a promising material for bone tissue scaffolds, due to their high biocompatibility, biodegradability, and mechanical properties.

Meng et al. [66] prepared a PVA/GO nanocomposite hydrogel as an artificial cartilage replacement using the freezing/thawing method by adding PEG. The obtained materials demonstrated improved mechanical strength and toughness, as well as increased dynamic stiffness for the sample with 1.5 wt% GO content. Moreover, the obtained hydrogel showed a superior lubrication effect, demonstrating a promising potential for cartilage replacement. Feng et al. [67] used chemical synthesis to prepare hydrogels based on CS and GO with injectability, self-healing ability, and adhesive properties for wound repair. Moreover, by varying the concentration of GO (0.1, 0.2, and 0.3 wt%), the mechanical and rheological properties of CS/GO hydrogels can be controlled. The in vivo experiments demonstrated hemostatic and wound healing capabilities in a rat liver bleeding model. PVA/GO hydrogels have been designed by a freeze–thaw method for use in biomedical and tissue engineering fields, but their poor mechanical and water-retention properties have hindered their development. Compared to pure PVA hydrogels, with the addition of 0.8 wt% GO, the tensile strength of GO/PVA hydrogels increased by 132%, suggesting an ideal load transfer between the GO and the PVA matrix. Also, adding different concentrations of GO does not affect the toxicity of PVA to osteoblast cells [68].

## 5. Photothermal Property of Graphene-Based Hydrogels and Their Biomedical Applications

Nanomaterials, such as graphene-based materials, have been widely used in medical applications due to their particular properties. The presence of oxygen functional groups (hydroxyl, carboxyl, epoxide, etc.) make the oxide sheets highly hydrophilic and enable GO to interact covalently or noncovalently with other materials forming stable aqueous colloids to ease the assembly of three-dimensional structures. The incorporation of GO materials into hydrogels not only improves the properties of hydrogels but also enhances their responses to external stimuli (e.g., pH, light, photothermal, electromagnetic, etc.). Moreover, the nature of nanomaterials decides the type and activity of stimuli-responsive hydrogels. Due to its extraordinary structure, GO can absorb light of different wavelengths, generating heat; thus, with the enhancement in photoabsorption, the photothermal capacity increases. Due to graphene’s extraordinary structure, it can strongly interact with low-frequency photons and generate heat under specific wavelengths such as NIR light through plasmonic photothermal conversion [69]. Thus, graphene-incorporated hydrogels are considered smart polymers because of their specific stimuli-absorbing properties, especially the high absorption of NIR light [70,71]. Graphene-based materials have been used in various applications, such as antibacterial and anticancer therapies, drug delivery, and tissue engineering. In this context, the most important factor is the photothermal conversion efficiency that depends on the material concentration, irradiation power, and time [72]. In the biomedical field, the development of smart hydrogels with properties such as photothermal properties, good mechanical properties, sustained drug release abilities, antibacterial properties, and biocompatibility is highly desirable [13]. Currently, graphene-based hydrogels are used in photothermal applications such as antibacterial and cancer therapies, tissue regeneration, and drug delivery. Photothermal graphene-incorporated hydrogels have the ability to destroy cancerous cells, inhibit tumors, and also can control drug release through photothermal effect. The release capacity of the drugs can lead to the killing of bacteria [19].

### 5.1. Cancer Photothermal Therapy

PTT has attracted widespread attention in cancer therapy due to its therapeutic efficiency: NIR radiation (applied to the affected area) has the capacity of penetrating tissue, allowing the release of the therapeutic drugs by increasing the local temperature [73]. In cancer therapy, when applying NIR irradiation, the produced temperature must be greater than 42 °C to cause hyperthermia in the damaged zone. When the tumors are located deep within tissues, this process becomes challenging. Thus, in order to increase the photothermal conversion, researchers started to use nanomaterials with strong absorption of NIR light [74]. The applications of graphene-based nanomaterials in PTT have been well studied because these materials exhibit remarkable NIR-absorbing capabilities. When applying NIR irradiation, the infrared radiation is absorbed by GO and transformed into thermal energy, and thus, the temperature in cell and tumor tissues increases, suppressing tumor development and destroying cancer cells [6]. The capacity of GO to deliver active agents at the tumor site is a promising strategy to increase the therapeutic potential of these nanomaterials.

**Figure 3:**
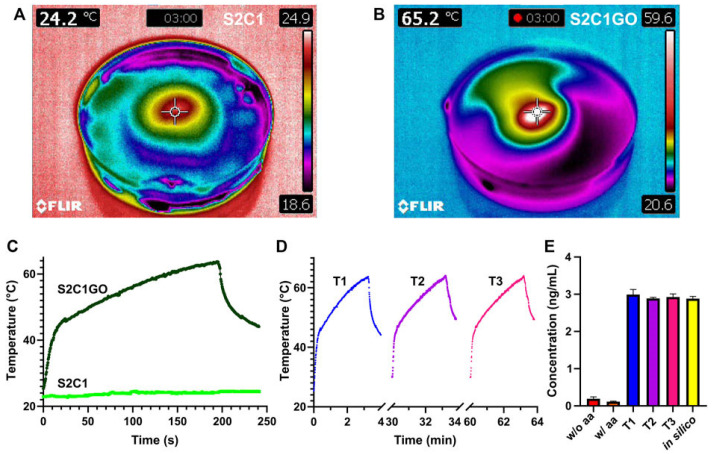
In vitro evaluation of the chemo/photothermal therapy. The temperature profile of (**A**) SISMA 2%/ChiMA 1% (S2C1) and (**B**) SISMA 2%/ChiMA 1%/graphene oxide (S2C1GO) hydrogels after 3 min irradiation with NIR. Photothermal heating of S2C1 and S2C1GO during (**C**) a first therapy cycle (T1) and (**D**) subsequent second (T2) and third (T3) therapy cycles. (**E**) Concentration of DOX released from S2C1GO for the different therapies using simulations (in silico) and unirradiated hydrogels with (w/) and without (w/o) ascorbic acid (aa) as positive and negative controls [77] (Copyright 2022 Creative Commons: Céspedes-Valenzuela, Sánchez-Rentería, Cifuentes, Gómez, Serna, Rueda-Gensini, Ostos, Muñoz-Camargo, and Cruz, doi:10.3389/fbioe.2022.947616).

In this regard, Li et al. [75] prepared self-healing hydrogels using chondroitin sulfate multi-aldehyde (CSMA), branched polyethyleneimine (BPEI), and BPEI-conjugated graphene (BPEI-GO) and explored its antitumor effect on breast cancer cells using chemo-photothermal therapy. The hydrogels improved sustained drug delivery, NIR-triggered photothermal effect, self-healing (~100%), and mechanical properties (7000 Pa). Moreover, the combination of DOX and PTT in CSMA/BPEI/BPEI-GO hydrogels reduced tumor recurrence by 66.7%. Liu and his collaborators designed a highly efficient NIR- and pH-responsive carboxymethyl CS- and rGO/aldehyde-functionalized PEG hydrogel, which exhibits a better photothermal conversion efficiency of 86.7% and a better drug release performance of DOX in the acidic environment (pH = 6.5) [76]. A recent study evaluated the in vitro photothermal antitumor activity on melanoma cell cultures of a CS methacrylate (CSMA)/porcine small intestine submucosa methacrylate (SISMA)/rGO-DOX hydrogel. The obtained smart therapeutic hydrogel was evaluated for its chemo/photothermal therapy, drug release, and biocompatibility, and the results revealed the capability of generating local hyperthermia and releasing DOX under NIR irradiation (Figure 3). The cytotoxicity tests demonstrated that, with the photo-induced release of DOX, cell death occurred, suggesting the cytotoxic effect of SISMA/CSMA/rGO-DOX smart hydrogel [77].

Nowadays, for cancer-related applications, injectable hydrogels have been receiving great attention because of their special properties. Injectable hydrogels (with a non-invasive procedure) allow local drug delivery into the diseased site, maximizing the accumulation of therapeutics in the tumor and reducing the risk of infection [26]. In this way, the formulation of GO-incorporated injectable hydrogels has been explored for cancer PTT. Lima-Sousa et al. [78] prepared thermo-responsive injectable hydrogels based on CS–garose incorporated with GO and rGO for cancer therapy and antibacterial activity. The obtained hydrogels demonstrated suitable injectability and gelation time. When NIR irradiation was applied, the breast cancer cells’ viability was reduced to 60% in case of thermogel-rGO and to 73% in case of thermogel-GO. When thermogels were incorporated with DOX/Ibuprofen, the formulation demonstrated a chemo-photothermal effect that further diminished cancer cells’ viability to 34%. Moreover, the antibacterial activity was increased when NIR laser irradiation was applied. A recent study demonstrated the in vivo and in vitro antitumor activities of berberine hydrochloride (BH) in situ thermo-sensitive hydrogel based on glycyrrhetinic acid (GA)-modified nano graphene oxide (NGO) (GA-BH-NGO-gel). The results showed an enhanced inhibition effect of tumor cells when laser irradiation was applied [79].

Moreover, to enhance the antitumor effects, the PTT technique has often been used in combination with other therapeutic approaches such as chemotherapy, radiotherapy, and gene therapy [80]. For example, a GO/CS thermosensitive hydrogel loaded with docetaxel (DTX) was developed by Zhu and collaborators. When NIR laser irradiation was applied, the DTX-GO/CS gel exhibited a higher antitumor efficacy in MCF-7 cells in vitro than that achieved without NIR irradiation. Furthermore, DTX-GO/CS demonstrated effectiveness in reducing tumor growth in tumor tissues of mice in vivo after 12 days of treatment [81].

### 5.2. Bacterial Killing and Wound Healing

Bacterial infections are attracting increasing attention because the long-term use of antibiotics leads to drug resistance, and thus, their treatment becomes complicated. Therefore, the use of PTT has started in this area, providing effective results in wound infections. Hydrogel-based dressings have been exploited as effective antibacterial materials, demonstrating various advantages in wound healing [82].

Liang et al. [83] developed injectable nanocomposite photothermal hydrogel dressings based on hyaluronic acid–graft–dopamine and rGO using a H_2_O_2_/HPR (horseradish peroxidase) system for wound regeneration. The obtained hydrogels exhibit good mechanical properties similar to human skin, high swelling and degradability, and antioxidant properties. Under NIR irradiation, the in vivo antibacterial behavior increased. A very low *E. coli* and *S. aureus* survival ratio (of 3.1%) was observed when the wound was covered with hydrogel and 10 min of NIR irradiation was applied. Furthermore, the hydrogel dressings confirmed the sustained drug release capacity and the improved collagen deposition, promoting wound closure. Han and his colleagues prepared dopamine-grafted gelatin (GelDA) and polydopamine-coated graphene oxide (pGO) for promoting wound repair. Adding pGO into the hydrogels leads to high photothermal antibacterial properties, which were demonstrated by both Gram-positive and Gram-negative bacterial tests against *E. coli* and *S. aureus*. After 15 min of irradiation, both *E. coli* and *S. aureus* did not survive. In vivo wound healing evaluation demonstrated the beneficial effect of hydrogel on promoting wound healing. After 14 days of treatment, the burn wound of the rat model was completely healed [84].

**Figure 4:**
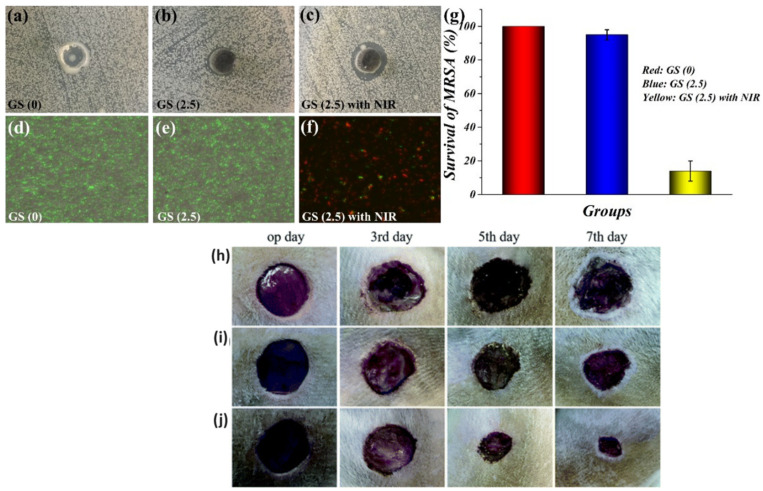
Antibacterial activity of GS hydrogels. Optical images of GS hydrogels with or without NIR laser irradiation against MRSA (**a**–**c**); live–dead fluorescent staining of MRSA (**d**–**g**). In vivo assessment of the GS hydrogels for wound healing. (**h**–**j**) Representative photographs of the skin wounds treated with PBS, GS (0), and GS (2.5) on day 0, day 3, day 5, and day 7. The scale bar is 500 mm [86] (Copyright 2021 Creative Commons: H. Zhang, S. Zheng, C. Chen, and D. Zhang, *RSC Adv.*, 2021, 11, 6367 DOI:10.1039/D0RA09106E, https://pubs.rsc.org/en/content/articlelanding/2021/ra/d0ra09106e, accessed on 5 December 2023).

A novel mesoporous polydopamine (MPDA)/GO/cellulose nanofibril (CNF) composite hydrogel loaded with tetracycline hydrochloride (TH) was obtained by Liu et al. Drug release experiments revealed a longer and controlled release in the case of MPDA@GO/CNF, compared to polydopamine/CNF hydrogel and pure CNF hydrogel, respectively. Moreover, by applying NIR light and changing the pH value, the rate of drug release could be accelerated. In vitro cytotoxicity tests revealed good biocompatibility, demonstrating that this material may have potential applications in wound healing therapies [85]. Zhang et al. [86] developed graphene/*N*-acryloyl glycinamide (NAGA) supramolecular hydrogels (GS hydrogels) with PTT for wound dressing applications. The physico-chemical properties showed high tensile strength (≈1.7 MPa) and good stretchability (≈400%). Moreover, it demonstrated significant antibacterial activity towards *S. aureus*. In vivo analyses showed improved skin tissue regeneration by applying NIR irradiation (Figure 4).

Zhang et al. [37] also evaluated the antibacterial activity of PVA/rGO/MoS_2_/Ag_3_PO_4_ composite hydrogel in wound infection under light irradiation. The in vitro antibacterial assay against *S. aureus* and *E. coli* under co-irradiation of 660 nm visible light (VL) and 808 nm NIR light within 10 min demonstrated excellent antibacterial properties. Also, the cytotoxicity assay showed good biocompatibility of the hydrogel.

Hydrogels based on natural polymers such as gel and alginate (Alg) are widely used for wound healing applications because of their structural resemblance to the ECM. Furthermore, human platelet-derived extracellular vesicles (pEVs) are known for their biological activity in healing diabetic wounds, due to their ability to modulate inflammation, cell proliferation, and angiogenesis. A recent study investigated the diabetic wound healing properties of a photothermal Gel/Alg loaded with rGO and pEVs. The Gel/Alg/rGO-pEV hydrogel demonstrated an increased drug release when NIR light was applied and the complete diabetic wound healing in streptozotocin-induced diabetic rat wound model. Furthermore, the Gel/Alg/rGO-pEV hydrogel promoted heat shock proteins expression implicated in cellular protective pathways [87].

### 5.3. Bone Tissue Regeneration

Graphene-based hydrogels are also studied for tissue engineering and bone regeneration. Bone cancer is one of the major diseases, representing a major threat to human health. Conventional therapies such as chemotherapy, radiotherapy, or surgical removal of the tumor tissue usually led to serious side effects. Thus, new therapies need to be developed with less drawbacks and the capacity to eliminate residual tumor cells and repair the bone defect site. In this regard, novel hydrogel composite scaffolds based on PVA/sodium alginate/hydroxyapatite (PVA/SA/HA) loaded with different concentrations of magnetic GO (MGO) and Fe_3_O_4_ nanoparticles were prepared and evaluated for bone repair and antitumor activity. The addition of MGO/Fe_3_O_4_ lead to enhanced physical and magnetothermal properties. The prepared hydrogel showed favorable anti-tumor characteristics and bone mesenchymal stem cell support [88].

Li et al. [89] designed a composite scaffold based on nano-hydroxyapatite (nHA) and rGO using the self-assembly method. The main scope of this research was to investigate if this three-dimensional hydrogel scaffold can be a desirable candidate for the treatment of large, tumor-related bone defects. The photothermal effect of nHa/rGO scaffold efficiently killed 92% of tumor cells (osteosarcoma MG-63) after 20 min of irradiation. The photothermal efficacy of the nHA/rGO scaffold was further analyzed in the animal experiments. After scaffold implantation, the tumor site was exposed to NIR irradiation and reached 60 °C after 4 min. The xenografted tumors growth was significantly inhibited, and the size of the tumors in the irradiated nHA-rGO group decreased. In addition, the scaffolds promoted the adhesion and proliferation of rat bone marrow stem cells (rBMSCs). In vivo experiments demonstrated that the nHA-rGO scaffold promotes bone regeneration in rat cranial defects. After the removal of osteosarcoma, common treatment such as implanting bone replacement materials can have disadvantages such as bleeding, promoting growing of residual tumor cells, or preventing soft tissue repair. Thus, Ma et al. [90] developed a new multifunctional scaffold, based on nHA/GO/CS cultured with MC3T3-E1 cells, for facilitating tumor cell death under NIR irradiation and promoting osteogenesis. The results revealed that reaching a temperature of 42 °C under irradiation could significantly promote cell proliferation, and by reaching a temperature of 48 °C, the obtained scaffolds could promote apoptosis and necrosis of tumor cells. A more recent study demonstrated the potential use of CS/rGO hydrogel film loaded with teriparatide (an FDA-approved drug) for osteoporosis treatment. The local delivery of the drug was achieved using NIR irradiation (at 808 nm, ~45 °C, 10 min/day, one week). In vivo studies showed new bone tissue formation through the biomimetic delivery of teriparatide in the osteoporotic rat model. Moreover, more blood vessels were observed between the newly formed bone and the center of bone defect [91].

### 5.4. Other Biomedical Applications

Apart from the above-mentioned applications, graphene-based hydrogels have been developed for use in other biomedical applications such as cell capture and release, treatment of diabetes, musculoskeletal inflammations, etc. Wang et al. [92] developed a new NIR-responsive GO/poly(*N*-isopropylacrylamide) (pNIPAM)/gelatin methacrylate (GelMA) hydrogel microcarrier system for controllable cell culture. To investigate the capture capacity of the microcarriers, HepG2 cells were used as model cells. The results showed an increased cell viability due to the incorporation of GelMA, photothermally responsive cell capture (less than 8% escaped from the microcarriers), and the capacity for proliferation and release due to the NIR absorption of GO. The in vivo experiments on immunodeficient mice demonstrated that NIR-responsive GO hydrogel microcarriers could facilitate tumor proliferation and angiogenesis.

Besides the drug delivery property, NIR light also has the capacity to enhance transdermal drug delivery (a pain-free alternative to hypodermic injections). Therein, a heat-active hydrogel based on GO or carboxyl-enriched rGO (rGO-COOH) loaded with metformin hydrochloride (an insulin agent used in the management of type 2 diabetes) was designed and investigated as a transdermal controlled release system. To demonstrate that metformin activity was unaffected by NIR irradiation, in vitro tests were performed by exposing Glucose-6 Phosphatase (G6P) in the human hepatocyte model to metformin at 45 °C, 55 °C, and 65 °C. In vivo assessment indicated the presence of metformin in mice plasma after 1 h of patch activation [93]. A thermo-sensitive hydrogel was designed by Mauri et al. using pristine graphene loaded with diclofenac (used for musculoskeletal and systemic inflammations) for several therapeutic treatments. By increasing the temperature, it was observed that a higher percentage of diclofenac was released (at 25 °C, a plateau was reached at 52%, whereas at 37 °C and 44 °C, the complete release was achieved after 6 h). Also, the cytotoxicity results demonstrated good biocompatibility, with an overall cell viability above 90%. These findings define these composite hydrogels as a promising tool for thermally triggered drug release in several healthcare scenarios [94]. NIR-responsive rGO/PEG dimethacrylate hydrogels (PEGDMA-rGOs) loaded with insulin were developed and studied for their applications in diabetic treatment. The hydrogel matrix provided sustained drug release upon NIR trigger at 980 nm and does not negatively impact the biological and metabolic activities of the released insulin. Moreover, cell viability did not decrease after irradiation, and the presence of the hydrogel did not induce any cytotoxicity in the Caco-2 epithelial cells. Thus, the obtained hydrogels have the ability to deliver insulin in a controlled manner using laser-induced photothermal activation. This insulin hydrogel should be of potential interest for the treatment of diabetes mellitus [95]. Due to their excellent physico-chemical properties, graphene-based hydrogels have been expanding towards biomedical areas, as summarized in Table 2. 

## 6. Conclusions and Future Perspectives

Over the years, graphene-based hydrogels have demonstrated remarkable structural (a high surface area, porosity, surface functionalization, and a tunable pore size distribution), functional (electrical conductivity, thermal conductivity, etc.), and mechanical properties (toughness, mechanical strength, and stiffness), making them highly attractive for a wide range of applications. Nowadays, photothermal-sensitive graphene-based hydrogels demonstrate a great potential in various biomedical and pharmaceutical applications, such as NIR-triggered drug release, anticancer therapy, antimicrobial therapy, wound healing, and bone regeneration. Numerous studies demonstrated their fascinating properties such as cost-effectiveness, functionalization, and a high photothermal conversion efficiency. In the present review article, the synthesis, photothermal-based biomedical applications, and recent advances of graphene-based hydrogels are discussed. Generally, in recent years, hydrogels have been widely studied and applied in the biomedical field due to their tunable physico-chemical properties and high biocompatibility, demonstrating their emerging potential for various disease treatments. Globally, the current applications of hydrogels focus on wound and eye diseases, diabetes, and neurological diseases (source: https://clinicaltrials.gov/database, accessed on 17 December 2023). Nevertheless, until now, there have been no clinical trials regarding the applications of GO-based hydrogel products, and thus, more efforts need to be made toward their lab-to-clinic transition. In order for GO-hydrogel products to move toward clinical applications, there are still some phototherapeutic challenges that need to be overcome/resolved, such as potential side effects and complications (e.g., inflammation, swelling, pain, fibrosis, calcification, etc.), real in vivo biotoxicity, thermal damage to normal tissues, insufficient therapy effect, and improper mode(s) of administration.

Scientific research demonstrated that photothermal GO hydrogels can inhibit the growth of bacteria and tumor cells by increasing the temperature generated by NIR irradiation and can control drug release. Therefore, scientists need to develop novel GO-based hydrogels with increased biocompatibility and physico-chemical properties for applications in photothermal biomedicine. Moreover, innovative strategies need to be developed to improve the practicality of PTT by modifying experimental parameters such as laser intensity, exposure time, and also the concentration of the photothermal agents for best results in the desired therapies. Using this strategy, smart hydrogels can be obtained that can be used in personalized therapies. In addition, GO-based hydrogels should be further explored in order to move forward towards preclinical studies. Another strategy that should be evaluated and studied more is the combination of PTT with conventional therapeutic strategies in cancer research, such as chemotherapy, radiotherapy, or/and immunotherapy. The scope of this approach is to achieve better therapeutic results by reducing the dose of PTT agents as well as their potential side effects. Recent studies demonstrated that the combination of chemotherapy with TPP can improve the chemotherapeutic effect of the anticancer drug in tumor tissue, and also the permeability of blood vessels, cell membrane, and ECM was improved [96,97]. In the present scenario, the focus should be directed towards the use of naturally active substances that have fewer side effects than the synthetic ones (that are usually used in cancer therapy); thus, researchers can make full use of the advantages of PTT, designing multifunctional structures of graphene-based hydrogels that can be suited for wider applications in the future.

To this date, scientific research has shown that nanomaterial-mediated PTT has emerged as an innovative approach, thus increasing the clinical utility of PTT. In summary, using these new-generation technologies combined with suitable PTT agents, there is a possibility of transferring these new PTT platforms from the laboratory to clinics.

## Figures and Tables

**Table 1:** Comparison of different preparation methods for graphene-integrated hydrogels [58,59].

Method	Advantages	Disadvantages
Physically crosslinked hydrogels	- Easy to obtain (one-step fabrication) and low cost; - Stable and uniform dispersion solutions; - Porous network structure and ultra-low density; - Biocompatibility, good absorption properties, great electrical and thermal conductivity, and stability.	- Poor mechanical properties due to crystallization, separation, and the irregular crosslinking dispersion of GO.
Chemically crosslinked hydrogels	- “Green” method, simple (one-step fabrication), and low cost; - Easy to obtain by the self-assembled ability of GO sheets; - High water retention capacity, biocompatibility, excellent electrical and thermal conductivities, and strong mechanical properties.	- Poor absorption properties, toxicity of the most commonly used reducing agents, and chemical moieties covalently bounded to the GO cannot be eliminated by washing steps.
In situ polymerization	- Easy to obtain and low cost; - Excellent pH sensitivity, swelling–deswelling ability, and strong interfacial interactions; - Favorable dispersibility of GO.	- Low stretching capacity and easily breakable at low deformation during elongation.

**Table 2:** Summary of the applications of hydrogels with PTT in different therapies.

Hydrogel	Agent	Application	Ref.
CSMA/BPEI/BPEI-GO	DOX	PTT/cancer therapy	[75]
CMC-rGO/CS-PEG	DOX	PTT/cancer therapy	[76]
SISMA/CSMA/rGO	DOX	PTT/cancer therapy	[77]
CS/agarose/GO CS/agarose/rGO	DOX/IBU	PTT/cancer therapy	[78]
GA/NGO	BH	PTT/cancer therapy	[79]
GO/CS	docetaxel	PTT/cancer therapy	[81]
DA/rGO	HA	PTT/wound healing/antibacterial	[83]
GelDA/pGO	mupirocin	PTT/wound healing/antibacterial	[84]
MPDA/GO/CNF	TH	PTT/drug release	[85]
NAGA/GS	-	PTT/wound healing	[86]
PVA/rGO	MoS_2_/Ag_3_PO_4_	PTT/wound healing/antibacterial	[37]
Gel/Alg/rGO	pEV	PTT/wound healing	[87]
MGO/PVA/SA/HA	-	PTT/bone regeneration/tissue repair	[88]
nHA-rGO	-	PTT/bone regeneration/cancer therapy	[89]
nHA/GO/CS	-	PTT/bone regeneration/tissue repair	[90]
CS/rGO	teriparatide	PTT/bone regeneration	[91]
GO/pNIPAM/GelMA	-	PTT/drug release	[92]
rGO-COOH	metformin	PTT/drug release/diabetes	[93]
PAA/FLG-HG	diclofenac	PTT/drug release	[94]
PEGDMA-rGO	insulin	PTT/drug release/diabetes	[95]

## Data Availability

No new data were created or analyzed in this study. Data sharing is not applicable to this article.
